# The Role of Nutritional Status on Polypharmacy, Cognition, and Functional Capacity of Institutionalized Elderly: A Systematic Review

**DOI:** 10.3390/nu13103477

**Published:** 2021-09-30

**Authors:** Catarina Caçador, Edite Teixeira-Lemos, Sofia Oliveira Martins, Fernando Ramos

**Affiliations:** 1 Faculty of Pharmacy, University of Coimbra, 3000-548 Coimbra, Portugal; cacasabel@hotmail.com; 2 ESAV, Polytechnic Institute of Viseu, 3504-510 Viseu, Portugal; etlemos3@gmail.com; 3 CERNAS-IPV Research Centre, Polytechnic Institute of Viseu, 3504-510 Viseu, Portugal; 4 Faculty of Pharmacy, University of Lisbon, 1649-003 Lisbon, Portugal; som@ff.ulisboa.pt; 5 Comprehensive Health Research Center, 7004-516 Evora, Portugal; 6 REQUIMTE/LAQV, University of Oporto, 4051-401 Porto, Portugal

**Keywords:** elderly, nutritional status, polypharmacy, cognition, functional capacity

## Abstract

Adequate nutritional status is necessary for the proper management of polypharmacy, the prevention of cognitive decline, and the maintenance of functional capacity in activities of daily living. Although several studies validate this fact for the general elderly population, data on institutionalized seniors concerning this relation are scarce. A systematic review was performed according to the PRISMA guidelines, aiming to study the potential correlation between nutritional status and polypharmacy, cognitive decline, and functional performance in institutionalized elders. The search was limited to studies in English or Portuguese in the last decade. Inclusion criteria relied on the PICO method. Five studies explored the relationship of nutritional status with cognitive performance in the institutionalized elderly, and nine prospective observational studies reported significant positive associations between appropriate nutritional status and physical abilities. Nutritional status was primarily measured by MNA. Adequate nutritional status was described as an important parameter in preventing cognitive and functional decline in the institutionalized elderly. No studies were found describing the impact of nutritional status on the prevention of polypharmacy. Given the strong impact of malnutrition found in the studies in cognition and functional abilities in the institutionalized elderly, an evaluation of nutritional status of the elders is crucial to prevent health problems and allow early intervention programs in order to further prevent health decline.

## 1. Introduction

Nutritional status is an important condition that deeply affects the general health of the elderly [1,2]. Despite the fact that the recommended intakes of most nutrients do not or only minimally change with age, decreased olfaction, taste, and vision combined with physiological changes that promote insufficient ingestion and poor absorption of essential nutrients may demand a compensatory nutrient intake as age increases [3,4].

While the worldwide population is increasingly aging and the number and proportion of the elderly in the overall population rise, the institutionalization of older adults is becoming an evident reality. This trend is driven by the increased demand for care of the elderly, whose families may not have financial or structural resources to support them [5,6]. The institutionalization process may radically affect the daily lives of older adults, namely in terms of nutrition, cognition, and level of functioning [7].

Among old, institutionalized subjects, alterations in nutritional status are frequently detected. The number of malnourished institutionalized elders is significant, ranging from 20% to 60%, depending on the criteria and methodology [8,9,10,11]. Moreover, this population is more vulnerable to depression, the use of anorexigenic drugs, and dependence on staff for feeding, which have been described as presenting a two- to three-fold increased risk for undernutrition [8,11].

Diseases which affect more than 80% of people over 85 years old may even increase drug consumption and the risk of polypharmacy [12,13]. Older people often present physiological changes related to aging that cause drug pharmacokinetics and pharmacodynamics changes. Hepatic elimination and renal excretion are particularly affected, interfering with the ingestion or absorption of nutrients, thereby increasing energy requirements [14]. Institutionalization presents an incremented risk factor over age for polypharmacy. Twenty to thirty percent of older adults take more than four medications, whereas nursing home senior residents take more than eight drugs per day [15]. Moreover, polypharmacy was observed to have a significant association with physical function, nutrition, and depression in the elderly [16,17].

Deficits caused by cognitive decline can lead to disability, thereby reducing and/or losing the ability to perform activities of daily living. Regular exercise and an active lifestyle were associated with a decreased risk of dementia [18,19,20,21]. Furthermore, functional and cognitive abilities and poorer nutritional status are reported to be very closely linked to each other. Malnourished older adults, or even those at risk of malnutrition, presented lower cognitive abilities than those with a normal nutritional status [22,23].

In the face of these numbers and facts, a relationship between nutritional status, polypharmacy, cognition, and functional ability in elders seems to be at first sight logical; however, concrete data are scarce and lack consistency in terms of both the tools and well-defined population characteristics used. The number of institutionalized seniors has increased worldwide, and only now are the first repercussions of this phenomena being reported [24]. The association between nutritional status and cognition, functional ability, and polypharmacy in the institutionalized elderly remains poorly characterized [25,26]. In this systematic review, we aimed to present a comprehensive overview of the peer-reviewed studies conducted specifically on nursing home residents, where nutritional status was correlated with polypharmacy, cognitive decline, and functional capacity. Nutritional patterns and needs are moldable factors at the individual level, and therefore this research may help to consolidate the relevance of the maintenance of nutritional status to preserve other health domains of the elderly that are institutionalized.

## 2. Materials and Methods

This systematic review was performed according to the relevant points of the PRISMA (Preferred Reporting Items for Systematic Reviews and Meta-Analyses) guidelines [27,28].

### 2.1. Eligibility Criteria

#### 2.1.1. Inclusion Criteria

Peer-reviewed studies describing interventional, observational, or randomized controlled trials were included. Inclusion criteria were established according to the above-described PICO strategy.

*Population:* Older adults over 65 years of age, living in nursing homes. No specific health condition was used for exclusion.

*Intervention:* All forms of nutritional patterns.

*Comparator/Control:* All studies were included irrespective of the presence or absence of comparator or control groups.

*Outcomes:* Any correlation between nutritional status with at least one of the other features (polypharmacy, cognitive function, and functional capacity). Notably, studies that assessed malnutrition by assessment tools (e.g., MNA) were included. There were no restrictions placed at the time of follow-up.

#### 2.1.2. Exclusion Criteria

Studies published in a language other than English or Portuguese.

Publications comprising editorials, comments, letters to the editor, guidelines, theses, books and scientific meeting abstracts, literature reviews, or case reports.

Studies conducted on participants with a mean age below 65 years and in a different setting than nursing homes.

Studies that did not report any results for an outcome measure of nutritional status.

Publications without description of the impact of nutritional status outcomes on polypharmacy, cognition, or functional ability.

Studies which used oral supplementation to preserve nutritional status. 

### 2.2. Search Strategy

In February 2021, an independent researcher (C.C.) searched the PubMed and Web of Science databases without language restrictions for the past ten years (since 2011). The author (C.C.) also reviewed the reference lists from the review articles reported in the PubMed and Web of Science searches to identify possible additional articles for inclusion. Cochrane library was also consulted; however, no additional studies were found. A combination of the following search terms was used: institutionalization AND Nutritional status AND Cognition, institutionalization AND Nutritional status AND functional capacity, institutionalization AND Nutritional status AND polypharmacy.

### 2.3. Selection Process

All search results were exported to Microsoft Office™ (Microsoft, Redmond, WA, US) Excel, using Mendeley Desktop® software (Mendeley, London, UK).

### 2.4. Data Extraction 

The following data were extracted from each study (CC) and validated by the second author (E.T.-L.), elaborating a systematic database:

Title, authors, and main aim of the intervention (cognitive/functional dependence/polypharmacy);

Demographic information of the participants: setting, country, sample size, sex, age;

Study characteristics: nature, aim;

Statistical analysis and outcomes;

Tools/methods used to collect data.

Outcome measures in the domains of nutritional status were sought independently or in combination with cases of decline in cognitive function and functional abilities. Differences in the criteria of assessment tools used for nutritional status, cognitive function, and functional abilities were recorded and discussed. 

Results from the initial search were evaluated separately by the two review authors (C.C. and E.T.-L.) according to the inclusion criteria. First, the results were screened by reading the article titles and excluding articles that were not relevant according to the inclusion criteria. Next, the study abstracts were evaluated, and non-relevant articles were excluded. Finally, the full-text articles selected by the two reviewers were collected and assessed for their relevance relative to the inclusion criteria. Any disagreements regarding the eligibility of studies were reconciled at the final step by discussion and consensus.

### 2.5. Risk of Bias (RoB) Assessment and Overall Quality

The methodological quality of the studies was assessed by two independent reviewers (C.C. and E. T-L.) based on different domains, such as study participation, confounding variables, measures of risk factors, analysis, and reporting. The risk of bias and the quality of each study were discussed between the two researchers until a consensus was reached. 

Evidence and methodological quality were assessed according to the Quality in Prognosis Studies (QUIPS) tool [29]. To rate the strength of study outcomes, the following six domains were considered: (1) Study Participation, (2) Study Attrition, (3) Prognostic Factor Measurement, (4) Outcome Measurement, (5) Study Confounding, and (6) Statistical Analysis and Reporting. Overall domain ratings were based on the number of assessment criteria in QUIPS met by each study in combination with their associated risk factors: if the majority of criteria were met with little or no risk of bias, a ‘++’ rating was assigned; if most criteria were met, but some flaws in the study posed an associated risk of bias, then a rating of a ‘+’ was assigned; and the domains in which most of the criteria were not met with significant flaws in key aspects of the study were given a rating of ‘-’.

Summarizing RoB is usually not linear, as there are no explicit criteria in the literature that pinpoint how to classify the overall RoB of a paper [22]. After continuous discussions from the authors, and after considering Study Participation, Prognostic Factor Measurement, and Outcome Measurement as critical to our review of the study, the following categorization was decided: (i) studies with a ‘++’ rating in at least two of the aforementioned critical domains were defined as low RoB; (ii) studies with a ‘-’ rating in any of the critical domains or with a ‘+’ rating in four or more domains were defined as high RoB; and (iii) all papers in between were classified as having moderate RoB. No article was excluded based on this assessment. 

## 3. Results

### 3.1. Study Selection and Literature Review

**Figure 1:**
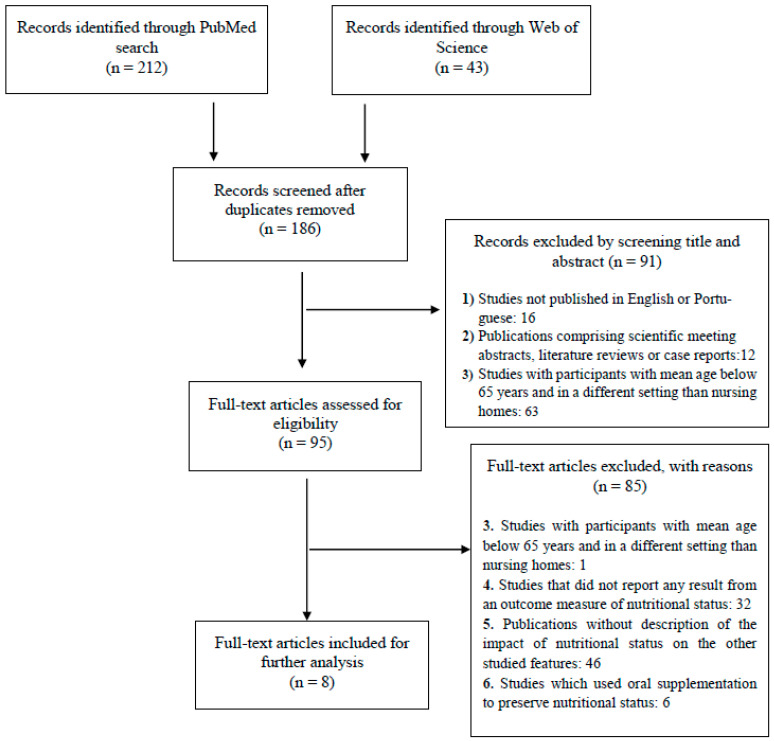
PRISMA flow diagram. Exclusion criteria were: (1) Studies published in a language other than English or Portuguese; (2) Publications comprising scientific meeting abstracts, literature reviews, or case reports; (3) Studies with participants with mean age below 65 years and in a different setting than nursing homes; (4) Studies that did not report any result from an outcome measure of nutritional status; (5) Publications without description of the impact of nutritional status outcomes in polypharmacy, cognition, or functional ability; and (6) Studies which evaluate oral supplementation efficacy.

The rationale for identification, screening, eligibility, and inclusion of articles is shown in Figure 1.

The search recorded 186 nonduplicated references, with 95 classified as potentially relevant after checking the titles and abstracts. After the screening of the full texts, 87 articles were excluded because they did not meet the inclusion criteria, namely the evaluation of nutritional status or its influence on cognitive or functional features. Ultimately, only eight original publications were selected and included in the review (Figure 1).

Table 1 displays a descriptive review of the included articles (n = 8), summarizing the impact of nutritional status on cognitive capacity and functional ability [8,30,31,32,33,34,35,36]. No studies were found to be associated with alterations in nutritional status and prevention of polypharmacy.

### 3.2. Literature Review

Table 1 displays a descriptive review of the included articles (n = 8), summarizing the impact of nutritional status on cognitive capacity and functional ability, respectively. No studies were found to be associated with alterations in nutritional status and prevention of polypharmacy.

### 3.3. Quality Assessment

Most of the studies were rated as having a low RoB (n = 5) based on the QUIPS tool in combination with the authors’ predefined criteria. These papers had strong study participation through methodologically validated tools in combination with clear descriptions of potential confounders and outcome measurements. Three studies were rated as having a “high” RoB (Table 2) [32,34,35]. The limitations identified in these studies were commonly considerable data loss and/or poor sampling frame and recruitment.

### 3.4. Participants and Follow-Up

Table 1 shows the number of participants assessed in each study included in this review as well as the mean age and the representativeness of females in the study samples. The final sample ranged from 23 to 2919. With the exception of Li et al., all of the samples included more than 60% of females. Follow-up periods varied considerably from 1 week to 5 years.

### 3.5. Characteristics of Studies and Outcomes Measures

Table 1 shows the methods used as outcome measurements in the included papers. Five studies [8,30,33,35,36] used MNA (short or long form) to evaluate nutritional status, which used a standard <17 points (long form) or <7 points (short form) as a measure of nutritional status. One study used BMI (kg/m^2^) to measure nutritional status [31], one used biochemical parameters, and another one used the geriatric nutritional risk index (GNRI) [34].

Cognitive function was measured in six studies [8,30,31,32,33,35]. The types of assessments differed slightly, with negligible variations in the cut-off value for the same type of assessment. Five studies [8,31,32,33,34,35] used the well-known and rapid Mini-Mental State Examination (MMSE). Two studies [32,33] defined <24 points as cognitive impairment. One study used <19 points as a measure of cognitive impairment [8], while another study [30] used the Short Portable Mental Status Questionnaire (SPMSQ) to define scores between 8 and 10 as intact cognitive functions. 

Seven studies [8,30,32,33,34,35,36] assessed functional capacity. Half of the studies (n = 4) [30,35,37] used the Barthel Index (BI), a tool developed to cover all aspects of self-care dependence in activities of daily living, where a score of 100 indicates functional independence. Notably, every study that used the BI reported cut-off values. Two studies measured the level of dependence on activities of daily living (ADL) [33,35], while one study [8] focused on ADL using the Katz Scale and assessed physical performance through the Short Physical Performance Battery (SPPB). One study assessed handgrip strength and used arm curl/lift as a performance test [32].

In all evaluated studies, a statistically significant association between cognition or functional capacity and nutritional status was pointed out. Serrano-Urrea and García-Meseguer [36] reported a positive association between MNA and BI scores (*r* = 0.375; *p* < 0.001), and Cereda et al. [34] concluded a significant association between GNRI and functional status.

Li et al. [30] found that both ADLs and depressive symptoms were significantly associated with nutritional status (*p* < 0.001). Similarly, Assis et al. [33] described that higher MNA scores (normal and at risk of malnutrition, MNA > 17) had higher scores in MMSE compared to malnourished ones (*p* < 0.001) and that more active participants who practiced between 9 to 13 ADLs had higher MMSE scores (*p* = 0.031) compared to those that practiced fewer activities. Donini et al. [8] validated the already reported results showing that physical performance, depression, and cognitive function were significantly and positively associated with the Man total score (*p* < 0.001). Pereira et al. [35] also associated nutritional status with cognitive capacity (*p* = 0.006), the suspicion of depression (*p* = 0.048), and functional capacity for ADLs (*p* < 0.001) as well as with dyslipidemia (*p* = 0.029).

Different blood biochemical parameters used to determine nutritional status were correlated with MMSE scores. Higher RBC folate and a lower tHcy concentration were associated with better global cognition as measured by the MMSE (*p* < 0.001) [31] as well acyl, HDL-cholesterol, ApoA, and albumin (*p* < 0.05) [32].

## 4. Discussion

Eight studies were found relating nutritional status with at least one of the two domains of cognition or functional capacity, and a clear relationship between nutritional status and cognitive and functional abilities was found in institutionalized seniors. 

According to the studies, a close relationship exists between nutritional and functional domains in long-term care residents [34,35]. Impairments in functional ability and nutritional status often occur with overlapping outcomes such as muscle loss, weakness, and frailty [38]. The results of this review found that there were slight variations in the assessment of functional abilities. The BI and the Katz Scale were the most commonly used tools to assess ADL in older adults. These two validated tools are very comprehensive and can provide useful insights into a patient’s functional capacity. The choice to use one of these tools is not often linear and should be made based on a case-by-case assessment. Notwithstanding, the Katz scale was developed to be recorded over a period of time. As such, it may be more suitable for long-term care settings. In addition, the approach to measuring functional dependence could benefit from a multifaceted strategy. Findings from this review suggest that anthropometric measures such as weight, height, waist circumference, and body mass index (BMI) may provide a more accurate understanding of functional status in older adults when combined with ADL assessment. 

Different studies have also shown that nutritional status affects cognition. Normal levels of folate, total homocysteine, serum cobalamin, HDL-cholesterol, and triglyceride levels were important biomarkers for cognition; however, they were not identified as predictive factors for cognitive decline [31,33]. Of note, Pedrero-Chamizo et al. also correlated these biomarkers with functional performance [32].

Depressive symptoms, and associated cognitive symptoms, are often reported in the institutionalized elderly. This fact is extremely relevant when evaluating the institutionalized elderly. In addition to presenting a higher risk of malnutrition, the prevalence of cognitive deficits and other neurological disorders is high among home care older adults. The findings of this review regarding the association between nutritional status and depression and subsequent cognitive performance are somewhat limited and should therefore be interpreted with caution. If preventive strategies fail to diagnose or treat depressive symptoms, specific dietary changes may be of immeasurable value. Nevertheless, the reported data are relatively limited. Therefore, further studies are needed to effectively understand the role of nutrition on this outcome. 

The findings of this review further demonstrate that there is little variation in the type of cognitive assessment, which in turn may partially contribute to the strength of some of the included studies. Notwithstanding, most of the included studies used the MMSE as one of the primary tools. One of the many advantages of this test is its ease of administration, despite the fact that it has been extensively criticized for its reliance on verbal interpretation [39]. This can eventually prove to be a major problem when administering the test to illiterate participants.

An effective relationship between nutrition and polypharmacy is yet to be consolidated [40]. Since certain diseases, per se, increase the likelihood of poor nutritional status, it is difficult to determine the independent role of drugs on nutritional status. Comorbidity-adjusted correlations show a strong link between nutritional status and excessive polypharmacy (more than nine drugs), whereas polypharmacy (six to nine drugs) has no association with nutritional status in non-institutionalized older adults [41]. To the best of our knowledge, no study has been conducted to correlate nutritional status with polypharmacy in the institutionalized elderly. Nevertheless, excessive polypharmacy was associated with declined nutritional status (*p* = 0.001), functional ability (*p* < 0.001), and cognitive capacity (*p* < 0.001) when compared to a non-polypharmacy group [41]. These data may eventually suggest that adherence to healthy dietary patterns could potentially delay the onset of age-related health deterioration and reduce the need for multiple medications. The support of pharmacists and physicians in nursing homes would also be of great value in maintaining strict control of medications. Therefore, the prescription of multiple drugs will be monitored to ensure minimal risk to the health of older adults.

Several instruments have been used to assess malnutrition or its associated factors. Although most studies used MNA to assess the nutritional status of the elderly, slightly different cut-off points and criteria were used, which might ultimately lead to over- or underestimation. Some easy-to-implement changes, such as increasing tea consumption, may have substantial results in psychomotor and cognitive-related tasks [40]. Considering that MNA does not measure the exact quantity or quality of fluid intake, further studies are needed to effectively understand the optimal type and quality of fluid intake in maintaining or improving the functional status of the elderly. 

The strengths of this review, in our opinion, are the low prevalence of high RoB studies and the broader understanding of the potential benefits of an adequate nutritional status on different outcomes, which, in our view, have not been adequately accounted for in the literature. However, most of the included studies were observational, thereby making it impossible to establish a cause-and-effect relationship. The fact that the clinicaltrials.gov database has not been included in the search strategy may eventually have contributed to the low rate of experimental studies included in the review; however, the prevalence of clinical trials in domains outside of medicine or similar is always very low, and some of them, if existing, could have been found in the searched databases. Additionally, the subjective nature of domain assessment is prone to bias. By independently reviewing the domain ratings, we hope to better adjust the overall quality scores of the included studies. The highly heterogeneous nature of nutritional interventions on different outcomes increases the complexity of the analysis. 

Our findings also include a few studies with low participation rates, which, when combined with variations in assessment methods, can eventually lead to inconsistencies that can hamper the reliability of the results.

In an aging society with an increasing number of institutionalized elders, this review highlights the urgent need for further research on the relation of nutritional status to functional capacity, cognitive status, and polypharmacy in the elderly population living in nursing homes. Randomized clinical trials would be the most suitable approach to generate robust results.

## 5. Conclusions

The eight studies included in this systematic review show that better nutritional status is associated with better cognitive function and functional capacity in the elderly. 

Since nutritional status can be adjusted and has been reported to have very intricate links to cognition, independence, and autonomy, a closer evaluation of nutritional status on these parameters is crucial to prevent associated health issues in the elderly population, especially the one that lives in nursing homes.

## Figures and Tables

**Table 1:** Description of reviewed studies on the impact of nutritional status on cognitive function and functional capacity in institutionalized seniors.

Author /Year	Study Design	Participants	Outcome Measurements				Main Results
			Nutritional Status	Cognitive Function	Functional Capacity	Other	
Li et al., 2013 [30]	Cross-sectional study	306 (Mean age: 80.6 ± 7.1, 47.7% female)	MNA	SPMSQ	Modified BI	NA	Both ADLs and depressive symptoms were significantly associated with nutritional status (*p* < 0.001).
Mendonca et al., 2017 [31]	Prospective longitudinal study Follow-up: 1.5, 3, and 5 years	765 (Age over 85 years old, 66.0% female)	BMI and Biochemical parameters: Baseline RBC folate, plasma vitamin B12, and tHcy concentrations	MMSE	NA	NA	Higher RBC folate and lower tHcy concentration measured at baseline were associated with better global cognition as measured by the MMSE (*p* < 0.001).
Donini et al., 2020 [8]	Cross-sectional study	246 (Mean age: 80.4 ± 10.5, 66.7% females)	Height, weight, and calf and mid-arm circumference measurements Modified MNA	MMSE	Katz Scale SPPB	Disease-related multi-morbidity: Cumulative Illness Rating Scale	Physical performance, depression and cognitive function ere significantly and positively associated with the M-MNA total score (*p* < 0.001).
Pedrero-Chamizo et al., 2020 [32]	Prospective longitudinal study Follow-up: 1 year	60 (Mean age: 80.6 ± 9.9, 68.3% females)	Biochemical parameters: Serum cobalamin, Total-cholesterol, HDL-cholesterol, LDL-cholesterol, triglycerides, apolipoprotein A1 (ApoA), apolipoprotein B (ApoB), lipoprotein A (LpA), glucose, albumin, and creatinine	MMSE	HGS, Upper body strength (Arm curl test), and Lower body strength (30 s chair stand test)	NA	MMSE scores showed a significant positive correlation with sCbl, HDL-cholesterol, ApoA, and albumin (*p* < 0.05). Significant negative correlations with HGS were observed for RBC folate, total cholesterol, LDL-cholesterol, and triglycerides. Biomarkers, except HDL-cholesterol lost their association with HGS when observed as covariates. HDL-cholesterol became the sole marker presenting a positive significant association.
Assis et al., 2020 [33]	Cross-sectional study	95 (Mean age: 73.3±12.5, 69.8% female)	MNA Anthropometric values: weight and height (to calculate body mass index—BMI), mid-upper arm circumference (AC), calf circumference (CC), waist circumference (WC), and hip circumference (HC)	MMSE	ADL	NA	The participants with higher scores in MNA (normal and at risk of malnutrition) had higher scores in MMSE compared to malnourished ones (*p* < 0.001). Participants that practiced more AADLs (9 to 13 activities) had higher MMSE scores (*p* = 0.031) compared to those that practiced fewer activities.
Cereda et al., 2013 [34]	Multicenter prospective cohort study Follow-up: 5 years	346 (Mean age: 85.7 ± 9.1, 74.6% female)	GNRI	NA	BI	NA	Functional status was significantly associated with nutritional risk by GNRI (*p* < 0.001).
Pereira et al., 2014 [35]	Cross-sectional study	359 (Mean age: 79.5 ± 9.3, 72.7% female)	MNA	MMSE GDS	Scale of ADL	Presence of comorbidity (hypertension, diabetes mellitus, and dyslipidemia)	Nutritional status was associated with dyslipidemia (*p* = 0.029), cognitive capacity (*p* = 0.006), the suspicion of depression (*p* = 0.048), and functional capacity for ADLs (*p* < 0.001)
Serrano-Urreaand García-Meseguer, 2014 [36]	Cross-sectional study	895 (Mean age: 82.3 ± 7.1, 58.4% female)	MNA	NA	BI	NA	MNA and the BI scores were positively associated (*r* = 0.375; *p* < 0.001)

ADL: Activity of Daily Living; BI: Barthel Index; BMI: Body Mass Index; GDS: Global Deterioration Scale; GNRI: Geriatric Nutritional Risk Index; HGS: Hand Grip Strength; MMSE: Mini-Mental State Examination; MNA: Mini Nutritional Assessment; *p*: *p*-value; *r*: Pearson Correlation value; SPMSQ: Short Portable Mental Status Questionnaire; SPPB: Short Physical Performance Battery.

**Table 2:** Overall risk of bias.

Study	1	2	3	4	5	6	Overall RoB Rating
Donini et al. [8]	++	+	++	++	-	+	Low
Li et al. [30]	++	+	++	++	-	+	Low
Mendonca et al. [31]	++	+	++	++	+	+	Low
Pedrero-Chamizo et al., 2020 [32]	+	+	++	++	+	+	High
Assis et al., 2020 [33]	++	+	++	++	-	+	Low
Cereda et al., 2013 [34]	+	-	+	+	+	+	High
Pereira et al., 2014 [35]	+	-	+	+	+	+	High
Serrano-Urrea and García-Meseguer, 2014 [36]	++	+	++	++	-	+	Low

1 = Study Participation; 2 = Study Attrition; 3 = Prognosis Factor Measurement; 4 = Outcome Measurement; 5 = Study Confounding; 6 = Statistical Analysis and Reporting; ‘++’ corresponds to low RoB, ‘+’ was assigned to moderate RoB studies, and ‘-’was given to high RoB.
